# Identification of Potential Candidate Genes From Co-Expression Module Analysis During Preadipocyte Differentiation in Landrace Pig

**DOI:** 10.3389/fgene.2021.753725

**Published:** 2022-02-01

**Authors:** Xitong Zhao, Huatao Liu, Yongjie Pan, Yibing Liu, Fengxia Zhang, Hong Ao, Jibin Zhang, Kai Xing, Chuduan Wang

**Affiliations:** ^1^ Beijing Shunxin Agriculture Co., Ltd., Beijing, China; ^2^ China Agricultural University, Beijing, China; ^3^ Chinese Academy of Agricultural Sciences, Beijing, China; ^4^ City of Hope National Medical Center, Duarte, CA, United States; ^5^ Beijing University of Agriculture, Beijing, China

**Keywords:** pig, preadipocyte differentiation, lipid metabolism, WGCNA, siRNA

## Abstract

Preadipocyte differentiation plays an important role in lipid deposition and affects fattening efficiency in pigs. In the present study, preadipocytes isolated from the subcutaneous adipose tissue of three Landrace piglets were induced into mature adipocytes *in vitro*. Gene clusters associated with fat deposition were investigated using RNA sequencing data at four time points during preadipocyte differentiation. Twenty-seven co-expression modules were subsequently constructed using weighted gene co-expression network analysis. Gene Ontology and Kyoto Encyclopedia of Genes and Genomes pathway enrichment analyses revealed three modules (blue, magenta, and brown) as being the most critical during preadipocyte differentiation. Based on these data and our previous differentially expressed gene analysis, angiopoietin-like 4 (*ANGPTL4*) was identified as a key regulator of preadipocyte differentiation and lipid metabolism. After inhibition of *ANGPTL4*, the expression of adipogenesis-related genes was reduced, except for that of lipoprotein lipase (*LPL*), which was negatively regulated by *ANGPTL4* during preadipocyte differentiation. Our findings provide a new perspective to understand the mechanism of fat deposition.

## Introduction

The Landrace pig is a globally adopted lean-type breed owing to its fast growth rate and lower fat content (Poklukar et al., 2020). Fat deposition is an important trait in pigs as it significantly influences meat quality, fattening efficiency, reproductive performance, and immunity (Verbeke et al., 1999). Hyperplasia and hypertrophy of the adipocytes are the two main processes affecting fat deposition (Kershaw and Flier, 2004; Choe et al., 2016). Pluripotent mesenchymal stem cells develop into adipoblasts and further into preadipocytes. Subsequently, preadipocytes differentiate into adipocytes under specific conditions (Gregoire et al., 1998). Adipocyte stromal vascular (S-V) cells from young pigs contain more preadipocytes capable of attaching and differentiating into mature adipocytes than those from older pigs (Akanbi et al., 1994). According to previous research, 7 days is an appropriate time to isolate S-V cells (Zhou et al., 2007). Multiple key regulators in adipocyte differentiation have been identified. For example, tumor necrosis factor-α (TNF-α) inhibits adipocyte differentiation (Cawthorn and Sethi, 2008), whereas CCAAT/enhancer-binding protein alpha (C/EBPα) regulates adipocyte terminal differentiation (Lane et al., 1999). Similarly, peroxisome proliferator-activated receptor gamma (PPARγ) can induce adipocyte generation (Ricote et al., 1998), whereas sirtuin 1 (SIRT1) can negatively regulate PPARγ to inhibit adipocyte differentiation (Picard et al., 2004). Adipose triglyceride lipase (Villena et al., 2004; Zimmermann et al., 2004), AMP-activated protein kinase (Yun and Zierath, 2006), SIRT1 (Cantó et al., 2010), perilipins (Greenberg et al., 1991; Londos et al., 1999), and hormone-sensitive lipase (Haemmerle et al., 2002) are involved in lipid metabolism.

Weighted gene co-expression network analysis (WGCNA) is a useful tool for exploring the complex relationships between genes and phenotypes in R (Langfelder and Horvath, 2008). WGCNA can transform gene expression data into a co-expression module that might be related to the phenotypic traits of interest (Horvath et al., 2006; Fuller et al., 2007; Shi et al., 2010; Udyavar et al., 2013). This method has been applied to various aspects of weighted correlation network analyses in studies on obesity (Morrison and Farmer, 2000; Kogelman et al., 2014; Kogelman et al., 2016). In pigs, WGCNA has been used to identify intramuscular fat-related gene sets (Zhao et al., 2020). Of note, gene interference has typically been used to determine gene function (Desaulniers et al., 2017; Chen et al., 2018).

Angiopoietin-like 4 (ANGPTL4), a secretory protein produced in the liver, kidneys, muscles, and adipose tissues (Singh et al., 2018), is a direct glucocorticoid receptor target that participates in glucocorticoid-regulated triglyceride (TG) metabolism (Koliwad et al., 2009). This protein is a strong regulator of lipid metabolism and obesity (Singh et al., 2018) and serves as an endogenous inhibitor of intestinal lipid digestion (Mattijssen et al., 2014). Although the regulatory role of *ANGPTL4* in lipoprotein metabolism is well established, its contribution to the regulation of pig preadipocytes and lipid deposition is not fully understood. In the present study, we found that *ANGPTL4* is associated with preadipocyte differentiation. We then determined its effect on preadipocyte differentiation using gene interference. Our study provides novel insights to understand the mechanism underlying fat deposition.

## Material and Methods

### Ethics Statement

All experimental procedures were performed following the Guide for Animal Care and Use of Laboratory Animals from the Institutional Animal Care and Use Committee at China Agricultural University. The experimental protocol was approved by the Department Animal Ethics Committee at China Agricultural University (Permit No. DK996).

### Isolation of Preadipocytes and Induction of Their Differentiation

Three Landrace piglets from a pig breeding farm in Ninghe (Tianjin, China) were obtained for the present study. The piglets were euthanized at the age of 7 days through an intraperitoneal injection of pentobarbital sodium (50 mg/kg body weight) and exsanguination. Subcutaneous adipose tissue samples were carefully resected under sterile conditions. The adipose tissue was digested with 0.1% type I collagenase (Sigma, Beijing, China) for approximately 2 h at 37°C and then centrifuged at 1,000 × *g* for 8 min (Zhou et al., 2007). The resulting digestion mixture was successively filtered through 100-µm mesh filters by centrifuging for 8–10 min to obtain preadipocyte pellets. The cell pellets were then resuspended in Dulbecco’s modified Eagle’s medium/nutrient mixture F-12 (DMEM/F12) containing 10% fetal bovine serum (FBS) and plated in glass culture dishes (Gibco, NY, United States). The culture medium was replaced every other day.

When the cells reached 90% confluence, they were transferred to six-well plates (Corning Costar, NY, United States) at a density of 3 × 10^6^ cells/ml in 2 ml per well, incubated at 37°C with 5% CO_2_ and 95% O_2_, and cultured until 90% confluence. The standard culture medium was then replaced with adipogenic induction medium (DMEM containing 10% FBS, 0.5 mM 3-isobutyl-1-methylxanthine, 1 µM dexamethasone, and 10 μg/ml insulin; Sigma) and cultured for 2 days. The differentiation medium was then replaced with maintenance medium (DMEM containing 10% FBS and 10 μg/ml insulin). Cell morphology was observed under a microscope. Twelve cell culture samples were obtained from each pig. The samples were collected from each pig at 0, 2, 4, and 8 days in triplicate and stored in liquid nitrogen until RNA isolation.

### Identification of Lipid Droplets

Oil Red O staining was performed to identify lipid droplets. The cell culture plates were gently washed with phosphate-buffered saline (PBS) three times and fixed in 10% formaldehyde for 15 min. The cells were then washed with PBS three times and stained with Oil Red O for 20 min. Finally, the cells were washed three times with PBS and photographed using an inverted microscope (Leica, Wetzlar, Germany). An equal volume of 100% isopropanol solution was added to the wells of each culture plate, and the absorbance at 500 nm was measured after thoroughly homogenizing the samples. Each experiment was repeated three times.

### RNA Isolation, Sequencing, and Sequence Data Processing

The total RNA was purified from the 12 samples using TRIzol reagent (Invitrogen, Carlsbad, CA, United States) according to the manufacturer’s instructions. RNA quantity and quality were assessed using the Agilent 2100 Bioanalyzer (Agilent, Santa Clara, CA, United States). All RNA samples with RNA integrity numbers >8.0 and an absorbance 260:280 ratio >1.9 were selected for library construction and deep sequencing.

From each of the 12 samples, 10 μg of RNA was used for RNA sequencing (RNA-seq) library preparation using the TruSeq® Stranded Total RNA Sample Preparation Kit (Illumina, CA, United States) according to the manufacturer’s instructions. The ligation products were size-selected by agarose gel electrophoresis and amplified by PCR. After purification and enrichment, 12 libraries were sequenced using the Illumina HiSeq 2500 system by Gene Denovo Biotechnology Co. (Guangzhou, Guangdong, China).

Raw reads were cleaned by removing adapter and primer sequences, reads containing more than 10% of unknown nucleotides (N), and more than 50% of low-quality (Q-value ≤ 20) bases. The clean reads were mapped to the pig reference genome (*Scrofa* 11.1, ftp://ftp.ensembl.org/pub/release-68/fasta/sus_scrofa/cdna) (Langmead et al., 2009) using Bowtie 2 (Langmead and Salzberg, 2012) and TopHat2 (version 2.0.3.12) (Trapnell et al., 2012) with default parameters. HT-seq (version 0.6.1) was used to calculate the read number mapped to each gene in each sample (Glaser et al., 2010).

Differential gene expression, based on the normalized read count of expressed genes, was analyzed in three time-point contrasts (0 vs. 2 days, 0 vs. 4 days, and 0 vs. 8 days) using the edgeR package (Robinson et al., 2009) in R (version 4.0.2). Genes with a false discovery rate (FDR) ≤0.05 and absolute log_2_ fold change (|log_2_FC|) >1 were identified as differentially expressed genes (DEGs) between two groups (Zhao et al., 2019).

### WGCNA

As mentioned previously, fat deposition is affected by preadipocyte differentiation. Thus, to identify candidate genes influencing preadipocyte differentiation, we applied WGCNA (Langfelder and Horvath, 2008) to fragments per kilobase of exon model per million mapped fragments (FPKM) values obtained from the RNA-seq of the 12 samples to identify genes associated with the degree of preadipocyte differentiation. Based on the expressed coding genes with FPKM >0, hierarchical clustering was performed. Outliers above a height threshold of 2,000 were filtered out. The remaining samples were used to establish an unsigned co-expression network.

We then used a one-step method to construct the network and determine the gene module. According to Zhang and Horvath (2005), gene co-expression networks should have scale-free characteristics and follow a power-law distribution. Hence, a weighted adjacency matrix was created, which can be defined as follows:

Aij = |cor × (xi,xj)|β, (1)

where *xi* and *xj* are the *i*th and *j*th genes, respectively.

Adjacent to the adjacent network is the combination of the soft-thresholding power parameter *β*, which is required to improve the co-expression similarity for computing the adjacency. To keep the network consistent with scale-free topology, the pickSoftThreshold() function in R was used to analyze its topology and select an appropriate soft-thresholding power value (*β*) to build it, and the mean connectivity of all genes in the module was evaluated. A power of 18 was selected as the soft threshold to ensure a scale-free topology network (*R* ^2^ = 0.85).

Subsequently, the topology overlap matrix (TOM) (Zhang and Horvath, 2005), which measures the connectivity of a pair of genes, was calculated from the adjacency matrix. Once the power value was determined, the TOM and dissTOM = 1−TOM were obtained. Based on hierarchical average linkage clustering using a dynamic tree-cutting algorithm, genes with similar expression patterns were clustered into the same modules and assigned a color (Zhang and Horvath, 2005).

After construction, the module eigengene (ME) was calculated using the first principal component of the expression profiles, which represented the weighted average expression profile. To identify biologically significant modules and select potentially critical modules for downstream analysis, the WGCNA approach was used to define the module–trait relationships (MTRs) (Kogelman et al., 2014) and gene significance (GS) of each module (Liu et al., 2016). We considered the time point of cell differentiation as a dichotomous variable (Langfelder and Horvath, 2008). A Pearson’s correlation test was run between the ME and time point of cell differentiation and between the expression profiles and time point of cell differentiation to estimate the MTRs and GS, respectively. The module significance (MS) was the mean GS value of the module genes. According to the selection criteria for critical modules reported in a previous study, modules with MTR > 0.30 and MS > 0.25 were considered candidate modules for functional enrichment analysis (Howard et al., 2017). Highly connected genes, also known as hub genes, may play important roles in a module (Wilhelm et al., 2004). Hub genes are relatively conserved at the core of the gene co-expression network, which can act as a genetic buffer to reduce the effect of other gene mutations (Kadioglu et al., 2021). Here, genes with GS > 0.3, module membership (MM) > 0.85, and intramodular connectivity > 5 were considered hub genes. Cytoscape (Shannon et al., 2003) was used to map the gene–gene interaction network for visualizing gene relationships.

### Functional Enrichment Analysis of Co-expression Modules and Selection of Candidate Genes

Gene Ontology (GO; http://www.geneontology.org/) is widely used in the field of bioinformatics to classify genes into terms from three different biological categories: cellular components, molecular functions, and biological processes. The default values were adapted for the parameters of the phenotype-related module, and three GO term enrichment analyses were performed on the genes in the module. Adjusted *p*-values <0.05 were considered significant, and the 10 most prominent entries for each analysis were retained.

Kyoto Encyclopedia of Genes and Genomes (KEGG, http://www.genome.jp/kegg/) is a database for systematic analysis of gene function and genomic information that facilitates the study of genes and gene expression as a part of an entire network. “ClusterProfiler” (Yu et al., 2012) and “ggplot2” packages were used to analyze and visualize such genetic information, respectively. The R package BioMart (http://www.biomarbiomart.org/) (Haider et al., 2009) was used to annotate genes in the module with Sscrofa11.1 as a reference genome. We selected a subset of modules based on their functional annotation and selected genes related to fat development. Based on the above information, the candidate genes affecting fat deposition were determined in this experiment.

### ANGPTL4 Knockdown

In our previous study (Zhao et al., 2019), *ANGPTL4* was identified as a DEG in all time-point contrast groups and was related to the PPAR signaling pathway and cell differentiation process. Similarly, in the present study, *ANGPTL4* was identified in a key module; therefore, we hypothesized that *ANGPTL4* has some regulatory effects on preadipocyte differentiation. According to the sequence of pig *ANGPTL4* (ID: 397,628) in GenBank, three pairs of siRNAs targeting and corresponding negative controls were designed and synthesized by GenePharma (Suzhou, Jiangsu, China) (Table 1). The siRNAs were centrifuged at 10,000 rpm for 2 min and dissolved in 125 μl of Nuclease-Free Tubes, Tips, and Buffers (DEPC) water (Gibco, NY, United States) to a final concentration of 20 μmol/L.

**TABLE 1:** siRNA sequences designed for *ANGPTL4*.

**Plasmid name**	**siRNA sequences**
si-752	5′-GGG​ACU​GCC​AGG​AAC​UCU​UTT-3′
	5′-AAG​AGU​UCC​UGG​CAG​UCC​CTT-3′
si-398	5′-GCA​UGG​CUG​CCU​GUG​GUA​ATT-3′
	5′-UUA​CCA​CAG​CCA​GCC​AUG​CTT-3′
si-1376	5′-CCC​UGC​UGA​UCC​AGC​CCA​UTT-3′
	5′-AUG​GGC​UGG​AUC​AGC​AGG​GTT-3′
Negative control	‘5′-UUC​UCC​GAA​CGU​GUC​ACG​UTT-3′
	5′-ACG​UGA​CAC​GUU​CGG​AGA​ATT-3′

Once the cell confluence reached 70%–80%, Lipofectamine 2000 (Invitrogen) was used for transfection. According to the manufacturer’s instructions, the medium was replaced with 2.5 ml of DMEM/F12 medium without serum and antibody, 30 min before transfection. Thereafter, 5 µl of Lipofectamine 2000 was diluted with 250 µl of serum and double-antibody-free DMEM/F12 medium per well, mixed gently, and incubated at room temperature (21°C–25°C) for 5 min; this was mixture I. Next, 10 µl of siRNA was collected from each well, diluted with 250 µl of serum-free and double-resistant DMEM/F12 medium, mixed gently, and incubated at RT for 5 min; this was mixture II. Mixtures I and II were gently mixed and allowed to stand for 20 min at RT. Thereafter, mixtures I and II were added to each well to a total volume of 500 µl and mixed thoroughly. After incubation at 37°C for 6 h, the medium was replaced with a complete medium. These steps were followed to induce cells and were repeated three times for each experiment.

### RNA Extraction and Quantitative Reverse-Transcription Quantitative Polymerase Chain Reaction

Based on our previous study (Zhao et al., 2019), *ANGPTL4* has the same expression pattern as Acetyl CoA acyltransferase 2 (*ACAA2*), aldehyde dehydrogenase two family member (*ALDH2*), and solute carrier family 27 member 1 (*SLC27A1*). Furthermore, lipoprotein lipase (*LPL*), stearoyl-CoA desaturase (*SCD*), and fatty acid synthase (*FASN*) are markers of fat deposition. Thus, we observed whether the expression of those genes changed before and after the interference of *ANGPTL4*.

The total RNA was extracted using a TRIzol reagent (Invitrogen) and reverse-transcribed into cDNA according to the manufacturer’s instructions. Reverse-transcription quantitative polymerase chain reaction (RT-qPCR) was performed using the Light Cycler 480 Real-Time PCR system (Roche, Hercules, CA, United States). The primers used for RT-qPCR detection of the selected genes are listed in Supplementary Table S1. All RT-qPCR experiments were performed using three biological replicates with three technical replicates for each sample. The 2^−ΔΔCt^ method was used to measure the change in mRNA abundance, and glyceraldehyde 3-phosphate dehydrogenase (*GAPDH*) was used as the internal control.

### Statistical Analyses

All qRT-PCR results are presented as mean ± standard deviation (SD). Statistical analyses were conducted using SPSS software (version 20.0; IBM Corp., Armonk, NY, United States); results with *p* < 0.01 were considered extremely significant, and those with *p* < 0.05 were considered significant.

## Results

### Phenotypic Changes During Preadipocyte Differentiation

**FIGURE 1:**
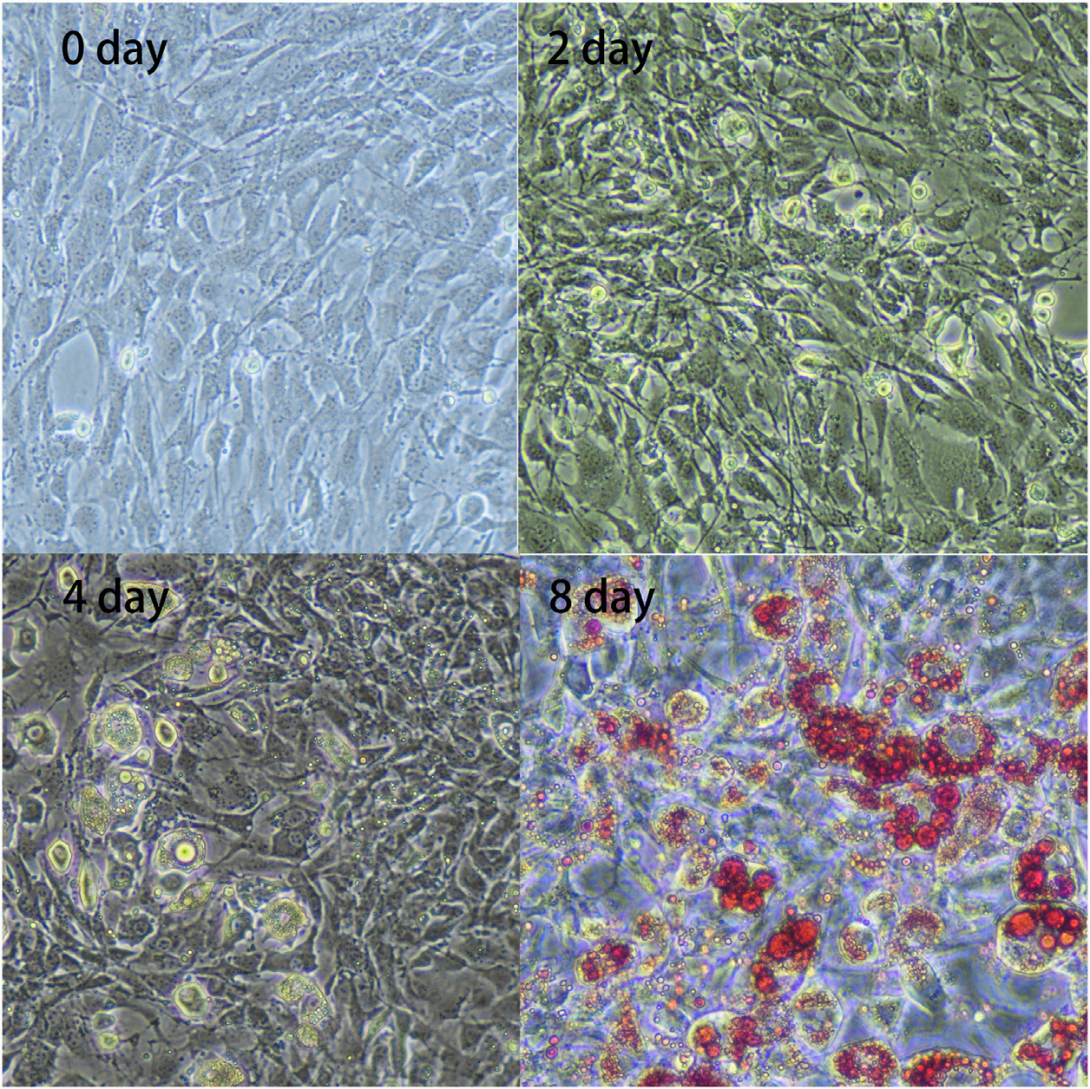
*In vitro* adipocyte differentiation. Preadipocytes were obtained from the subcutaneous adipose tissue of three 7-day-old Landrace pigs and were cultured and collected at four differentiation stages: 0, 2, 4, and 8 days. The figure shows enlarged representative photographs of adipocytes obtained during differentiation [day 0, 2, 4, and 8; day 8 with Oil Red O staining (×20)].

Compared to cell shapes observed during the initial phase (day 0), preadipocytes gradually changed from fibrous to spherical on day 2. Subsequently, lipid droplets became visible on day 4, gradually increasing in number until day 8 (Figure 1). These results indicated that the preadipocytes differentiated successfully and could be used for further analysis.

### Construction of Co-expression Modules

Genes with a sum of FPKM values >0 (*n* = 12,816) were selected for a co-expression network analysis (Supplementary Table S2). The WGCNA package in R was used to construct co-expression modules. No outlier samples were found in the hierarchical clustering of samples using the flashClust package in R (Supplementary Figure S1).

**FIGURE 2:**
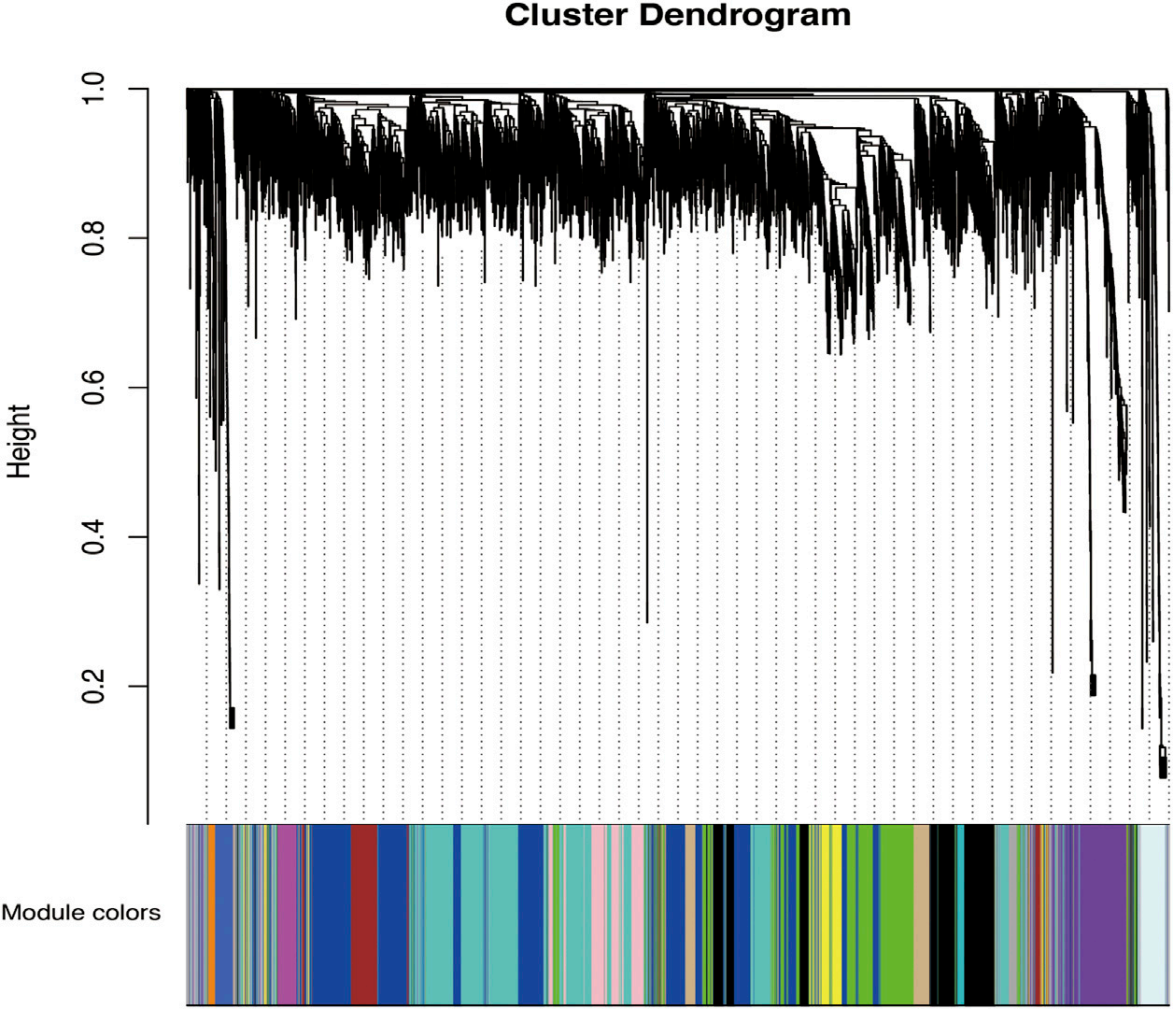
Categorization of gene modules. The figure shows the clustering of genes, and the categorization of gene modules based on clustering. Branches of the same color were categorized into the same gene module. From our analysis, 27 co-expression modules were constructed and are shown in different colors here. These modules ranged from large to small based on the number of genes they included. The number of genes in each module is presented in Table 3.

According to the standard of a scale-free network, we selected an appropriate weighted parameter of the adjacency function, namely, the soft threshold, which was 18 in this study (Supplementary Figure S2). We then calculated the correlation and adjacency matrices and combined them into the topology matrix. We finally identified 27 gene modules based on genetic similarity after merging modules with dissimilarities less than 0.25 and a minimum size of 30 (Figure 2; Supplementary Table S3).

### Analysis of the Relationship Between Gene Modules and Preadipocyte Differentiation

**FIGURE 3:**
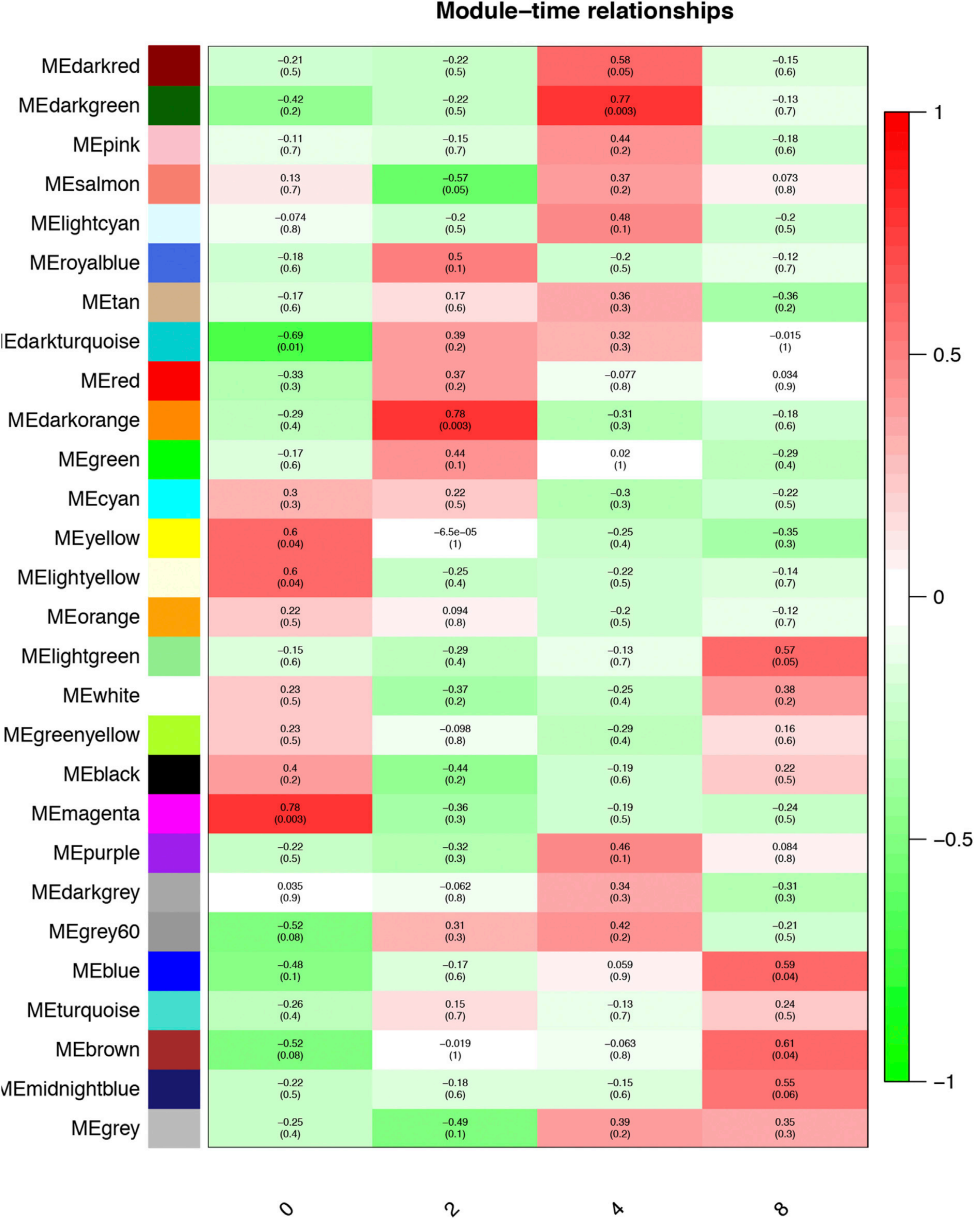
Module–time correlation. Correlation between gene modules and sample information, with the x- and y-axes representing preadipocyte differentiation time points and gene modules, respectively. The darker the color, the higher the correlation, with red and green representing positive and negative correlation, respectively. The *p*-value is enclosed in brackets.

The Pearson correlation coefficient between the eigengenes of modules and the corresponding variables represents the linear correlation between the module and phenotypic information (Figure 3). We found that the blue and brown modules were significantly correlated with day 8 (*r* = 0.59, *p* < 0.05; Figure 3), which was the time point with the most preadipocytes, suggesting that genes in these two modules promote preadipocyte differentiation. In addition, the magenta module was significantly correlated with day 0 when there was little differentiation (*r* = 0.78, *p* < 0.01), suggesting that these genes inhibit preadipocyte differentiation.

**FIGURE 4:**
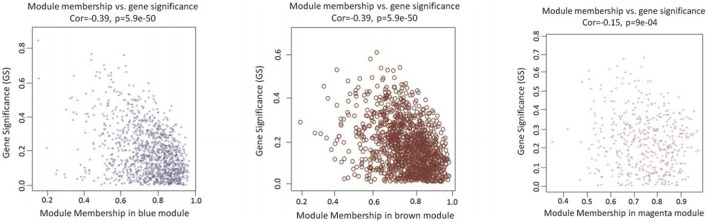
Scatterplots of GS vs. MM in candidate modules. The figure shows the GS in the three significant modules correlated with adipocyte differentiation. The x-axis represents the value of membership in each module, and the y-axis represents the GS of the genes in the blue, brown, and magenta modules. The gene in the lower right corner of each graph is the hub gene that is of interest to us. These genes are highly correlated with phenotypes and have a high MM, which is a good representation of the gene module.

As a final module assessment, scatter plots of GS for preadipocyte differentiation versus MM for the blue, brown, and magenta modules are presented in Figure 4. We found a significant correlation between GS and MM, suggesting that genes in the preadipocyte differentiation-related modules tended to highly correlate with fat deposition.

### Functional Enrichment Analysis of Genes in Candidate Modules

For the genes in the blue, brown, and magenta modules, GO term and KEGG pathway enrichment analyses were performed (Table 2, Table 3). We found that genes in the blue module play a role in fatty acid degradation, those in the brown module play a role in mitogen-activated protein kinase (MAPK) signaling pathways, and genes in both modules participate in fatty acid beta-oxidation. Genes in the magenta module are related to fatty acid beta-oxidation using acyl-CoA dehydrogenase, the Wnt/PI3K-Akt/TGF-beta signaling pathway, and the regulation of stem cell pluripotency. These results suggested that genes in the blue, brown, and magenta modules are related to lipid metabolism.

**TABLE 2:** GO terms of candidate modules (adjusted *p*-value ≤ 0.05).

Module	Term ID	Term name	*p*-value	Genes
Blue	GO:0006635	Fatty acid beta-oxidation	2.04E−02	ECI1, ACAA2, PEX5, ABCD3, BDH2, ACAT1, ACAA1, ANGPTL4
	GO:0000062	Fatty-acyl-CoA binding	2.78E−02	GCDH, ECI2, ACADSB, ACADS, ACBD6, ACBD4
Brown	GO:0006635	Fatty acid beta-oxidation	1.67E−02	ECHDC2, HIBCH, HSD17B4, SESN2, CROT, ACOX3, AUH
Magenta	GO:0033539	Fatty acid beta-oxidation using acyl-CoA dehydrogenase	1.11E−02	ACADVL, ETFDH, ACAD9, ETFB
	GO:0090263	Positive regulation of canonical Wnt signaling pathway	2.37E−02	DKK2, CAV1, SFRP2, COL1A1, LRRK1, SRC

**TABLE 3:** KEGG analysis of candidate modules (adjusted *p*-value ≤ 0.05).

Modules	Term	*p*-value	Genes
Blue	cfa00071:Fatty acid degradation	2.05E−04	ECI1, GCDH, ECI2, ACAA2, ACADSB, ALDH7A1, ACADS, ACAT1, ACSL3, ALDH9A1, ACSL5, ACAA1
Brown	ptr04010:MAPK signaling pathway	5.15E−03	FGFR1, IL1R1, FGFR4, MAP4K2, CACNB1, MAPKAPK3, MKNK1, FOS, JUND, MAP3K8, PPP3CC, PRKACB, MYC, TAOK2, CACNG7, CACNG5, MAPK10, TAB1, DUSP5, NRAS, DUSP1, JUN, NTRK1, GADD45G, GADD45B, MAP3K12, DUSP7, DUSP6
Magenta	cfa04151:PI3K-Akt signaling pathway	2.76E−02	FGF19, IBSP, FGF18, FGF5, IL7, PDGFA, COL3A1, COL5A2, COL5A1, SOS1, TNR, COL6A2, COL1A2, LAMC1, COL1A1, PIK3R3, LAMB1
	cfa04350:TGF-beta signaling pathway	2.61E−02	INHBA, GDF6, CREBBP, BMPR2, TGFB3, BMPR1B, ACVR1
	cfa04550:Signaling pathways regulating pluripotency of stem cells	5.11E−03	WNT10A, INHBA, WNT16, BMPR2, WNT9A, PIK3R3, BMPR1B, WNT6, MEIS1, TCF3, ACVR1

### Module Visualization and Hub Genes

**FIGURE 5:**
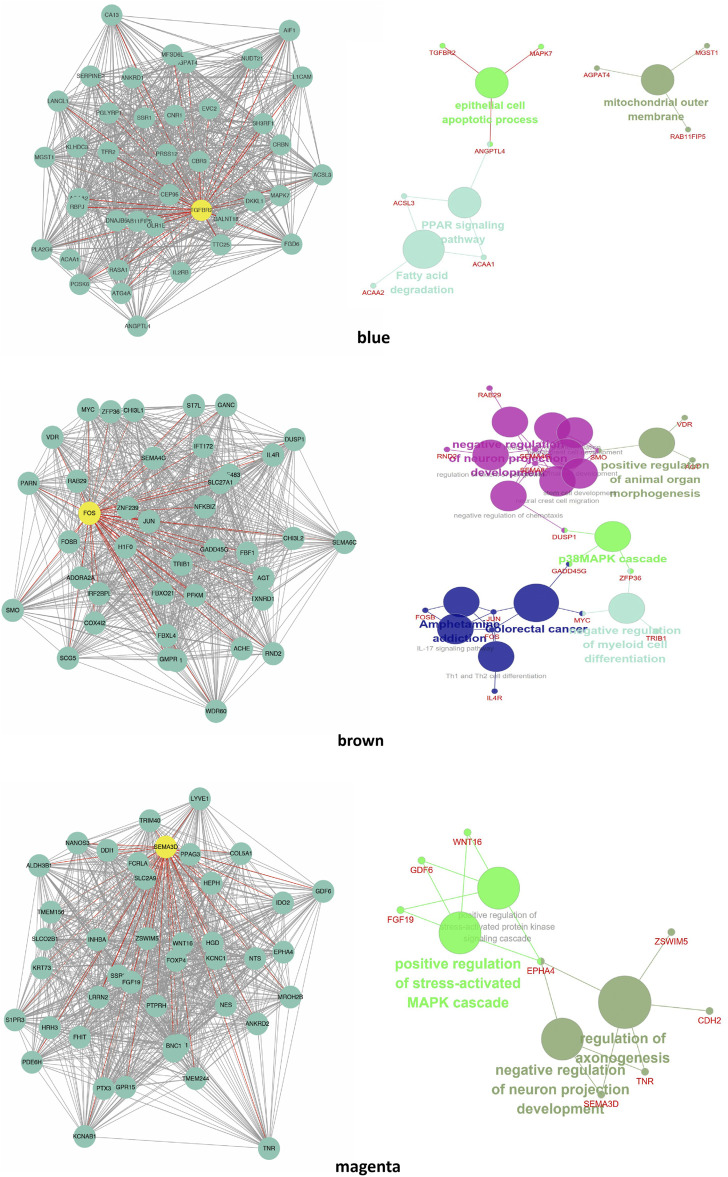
Visualization of modules. The network diagram is shown on the left, and the enrichment analysis is on the right. The hub genes in the modules are bold in yellow.

Genes with the highest degree of connectivity were selected from the three candidate modules, and Cystoscape software was used to draw the gene interaction network diagrams (Figure 5). We found *ANGPTL4* in the blue module (Supplementary Table S4). Thus, we knocked down *ANGPTL4* to observe its effect on preadipocyte differentiation.

### Transfection Efficiency of siRNAs and Analysis of the mRNA Expression of the Related Genes

**FIGURE 6:**
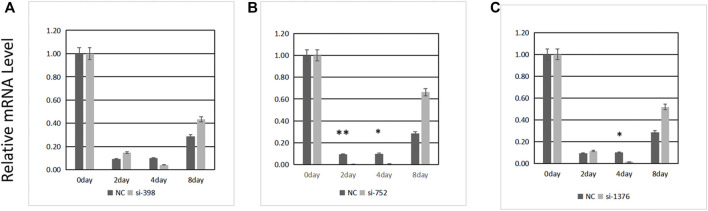
Transfection efficiency of three siRNAs. **(A)** si-398, **(B)** si-752, **(C)** si-1376. *Significant (*p* < 0.05), **extremely significant (*p* < 0.01).

Three pairs of siRNAs were synthesized. The expression level of *ANGPTL4* was determined 0, 2, 4, and 8 days after differentiation following transfection (Figure 6). The transfection efficiency of siRNA-752 was the highest (>90%); thus, it was used in the subsequent experiments.

**FIGURE 7:**
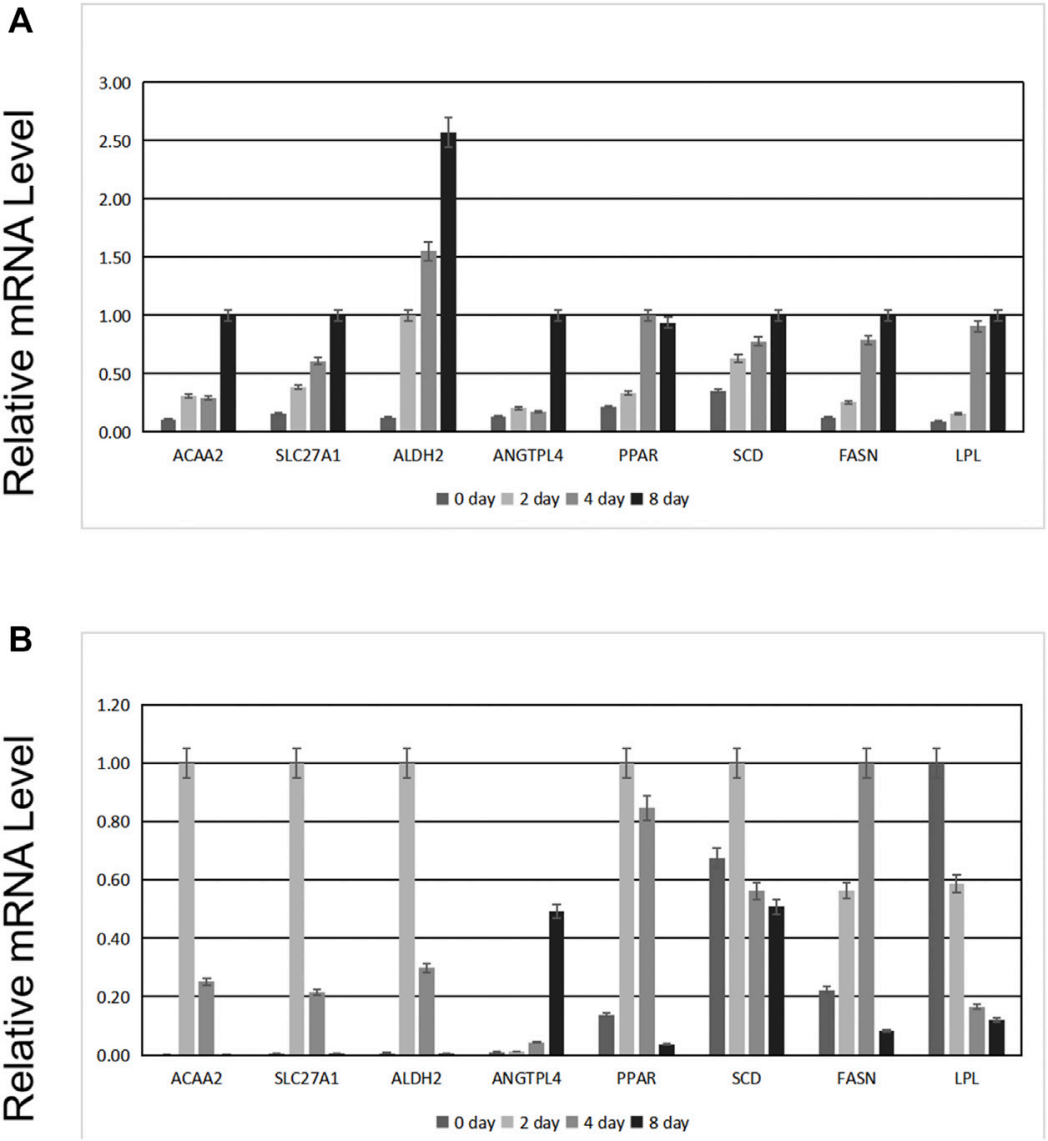
Effects of *ANGPTL4* knockdown on the mRNA expression levels of the related genes. **(A)** Expression before transfection of siRNA. **(B)** Expression after transfection of siRNA.

As shown in Figure 7A, the mRNA levels of *ANGPTL4*, *ACAA2*, *SLC27A1*, *ALDH2*, *PPAR*, *SCD*, *FASN*, and *LPL* increased with preadipocyte differentiation. After transfection, we observed changes in the expression levels of these genes. The expression level of *ANGPTL4* significantly decreased, confirming successful transfection. Except for *LPL*, whose expression level was opposite to that of *ANGPTL4*, the expression of the other genes initially increased and then decreased (Figure 7B).

### Effects of *ANGPTL4* Knockdown on the Differentiation of Pig Preadipocytes

**FIGURE 8:**
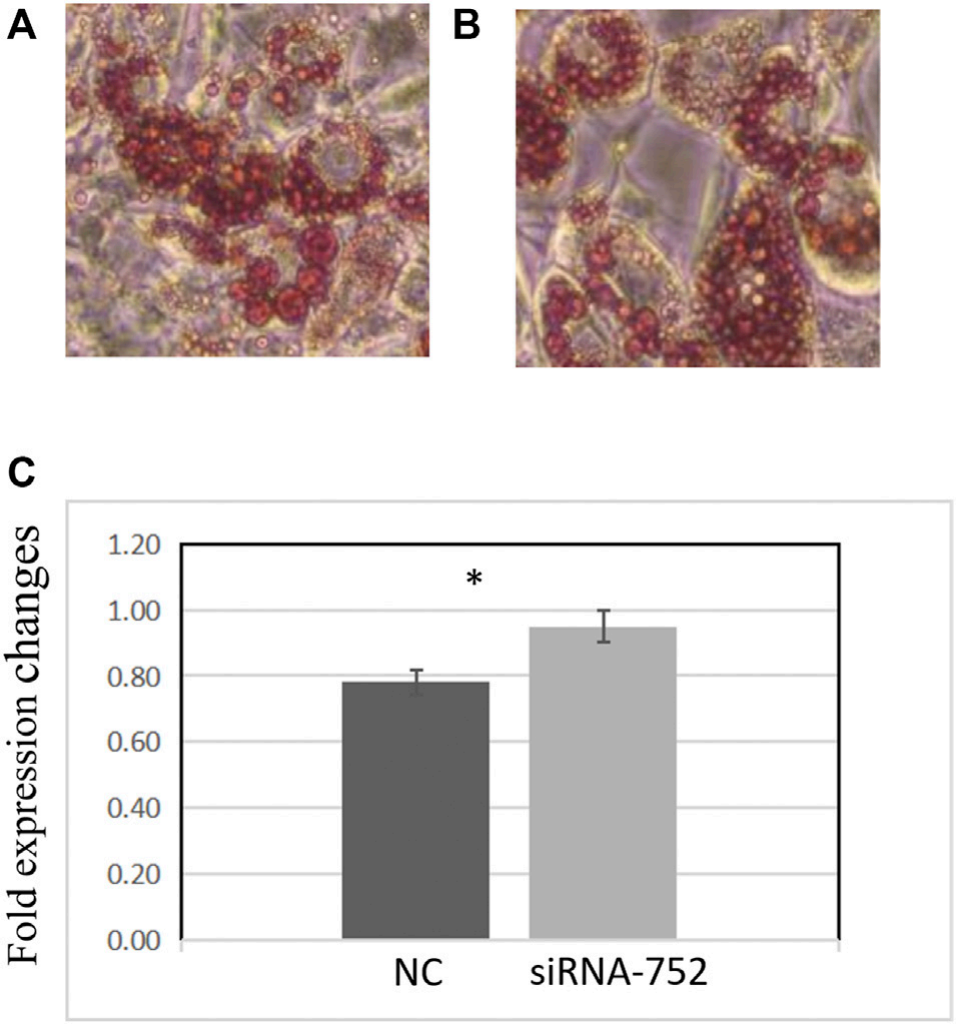
Effects of *ANGPTL4* knockdown on the differentiation of pig preadipocytes (day 8). **(A)** Adipocyte differentiation in the negative control group (×20). **(B)** Adipocyte differentiation in the siRNA-752-treated group (×20). **(C)** Optical density (OD) values at 500 nm after Oil Red O staining. Data are presented as mean ± standard deviation. *Statistically significant differences (*p* < 0.05, *n* = 3).

Two days after transfecting preadipocytes with siRNA-752, the inducer was added to promote differentiation. The cells were then stained with Oil Red O and observed under a microscope. Compared with the negative control group, the siRNA-752 transfection group showed a significant increase in lipid droplet production (Figures 8A,B) and OD_500nm_ values (*p* < 0.05) (Figure 8C), indicating that the knockdown of *ANGPTL4* promoted the differentiation of porcine preadipocytes.

## Discussion

Lipid metabolism is a pivotal factor in maintaining health and determining fat deposition (Mourot and Hermier, 2001). However, given the complex and dynamic nature of lipid metabolism regulation (Kahn and Flier, 2000), we do not yet fully understand the role of genes in this process. In the present study, we performed WGCNA using the RNA-seq data of Landrace preadipocytes to identify fat deposition-related genes.

We constructed 27 co-expression modules with 12,816 genes from 12 pig preadipocyte samples collected at four time points using the WGCNA method to identify genes involved in preadipocyte differentiation. WGCNA offers several distinct advantages over other methods; for instance, it allows an analysis to focus directly on the association between co-expression modules and lipid metabolism (Shubham et al., 2017; Hu et al., 2018; Li et al., 2018). Genes in the same module are generally connected to the same terms of function. Therefore, the analysis enabled the identification of biologically relevant modules and hub genes that may eventually serve as biomarkers for diagnosis or treatment.

Three modules were the most closely related to preadipocyte differentiation. The blue and brown modules were significantly correlated with day 8 and may be related to lipid deposition. Conversely, the magenta module was significantly correlated with day 0 and may play an inhibiting role in adipocyte differentiation. The GO term and KEGG pathway enrichment analyses showed that the blue module was related to fatty acid beta-oxidation, fatty-acyl-CoA binding, and fatty acid degradation. The brown module was related to fatty acid beta-oxidation and MAPK signaling pathways, which are activated in response to a variety of extracellular stimuli, such as growth factor stimulation, and play an important role in lipid localization (Anderson, 2006). The magenta module was found to be involved in the positive regulation of the canonical Wnt signaling pathway. Wnt/β-catenin signaling is the central negative regulator of adipogenesis (Ross et al., 2000; Bennett et al., 2002) and is related to lipid accumulation (Tian et al., 2019). Additionally, the magenta module was enriched in signaling pathways regulating the pluripotency of stem cells, which might be related to preadipocyte proliferation. These findings suggest that the expression of genes in the blue and brown modules increased with hypertrophy, whereas the genes in the magenta module contributed to hyperplasia.

We then identified hub genes in the three co-expression modules. The hub gene in the magenta module was semaphorin 3D (*SEMA3D*), which promotes cell proliferation (Berndt, 2006) and may be involved in regulating cell sorting (Lallier, 2004). The hub gene in the brown module was the Fos proto-oncogene AP-1 transcription factor subunit (*FOS*), which belongs to the JUN family and is a regulator of cell proliferation, differentiation, and transformation (Karin et al., 1997; Shaulian and Karin, 2001). *FOS* may also be related to lipid homeostasis (Izawa et al., 2015). These hub genes are potential biomarkers for preadipocyte differentiation and lipid metabolism and require further research. The hub gene of the blue module was transforming growth factor-beta receptor 2 (*TGFBR2*), which is related to cell apoptosis and cell cycle arrest (Oft et al., 1998). *TGFBR2* may influence lipid metabolic activities through TGF-β signaling (Iwata et al., 2014). In addition, *ANGPTL4* and *ACAA2* in the blue module were found in the same cluster as that in our previous study (Zhao et al., 2019). *ACAA2* is a key enzyme in the fatty acid oxidation pathway that catalyzes the last step of mitochondrial beta-oxidation, thus playing an important role in fatty acid metabolism (Sodhi et al., 2014; Wang et al., 2016). Beta-oxidation of fatty acids may affect muscle fat content by changing the content of fatty acids, which may ultimately affect meat quality. Similarly, a previous study reported that *ACAA2* promotes sheep preadipocyte differentiation (Zhang et al., 2019) and is related to swine fat deposition (Xing et al., 2015). *ANGPTL4*, which is in the same module as *ACAA2*, may have similar functions.

In this study, we inhibited *ANGPTL4* expression and observed the changes in the mRNA levels of related genes. We found that although fewer fat droplets were produced, the process of adipocyte differentiation was still completed, suggesting that lipid metabolism is a complex process influenced by multiple factors and is not offset by a single gene. However, it is important to note that we interfered with gene expression with an approximate efficiency of 90% and did not completely knock out *ANGPTL4*. Therefore, it is possible that trace amounts of the target gene product continued to play a role. This is a limitation of this study that needs to be addressed in future studies.

After the inhibition of *ANGPTL4* expression, only the expression trend of *LPL* was different from that of other genes. This may confirm the suggestion of several studies that *ANGPTL4* negatively regulates *LPL*. Plasma TG levels are primarily determined by the dynamic balance between TG absorption in the small intestine and TG degradation in muscles and adipose tissues (Rosen and MacDougald, 2006). LPL is particularly important as a rate-limiting enzyme for the hydrolysis of TGs in the blood (Yagyu et al., 2003). This enzyme is produced by muscle cells and adipocytes, transported to the surface of endothelial cells after binding with glycosylphosphatidylinositol-anchored high-density lipoprotein binding protein 1 (GPIHBP1) (Davies et al., 2010), and primarily regulated at the post-translational level (Ogata and Oku, 2001). ANGPTL4, as an equilibrium switch that regulates homeostasis, increases blood TG levels by inhibiting LPL activity (Yoshida et al., 2002). ANGPTL3 and ANGPTL8, which are members of the same angiopoietin-like protein family as ANGPTL4, might also participate in the regulation of *LPL,* and their coordination ensures the balance of TG levels. ANGPTL3 and ANGPTL4 are inhibitors of LPL activity, and ANGPTL8 inhibits LPL secretion (Kovrov et al., 2019). During exercise, fasting, and cold exposure, ANGPTL4 inhibits LPL activity in the adipose tissues, skeletal muscles, and heart; inhibits LPL-mediated circulating TG clearance; reduces the entry of plasma TG-derived fatty acids into adipose tissues; and promotes their absorption by oxidized tissues (Kroupa et al., 2012; Catoire et al., 2014; Dijk et al., 2015). However, the inhibition of LPL by ANGPTL4 is rapidly eliminated after the stable binding of GPIHBP1 with the dimer form of LPL (Sonnenburg et al., 2009). Fasting reportedly induces higher expression of ANGPTL4 in the adipose tissues, and it reduces LPL activity by promoting protease lysis of LPL in cells (Dijk et al., 2018). ANGPTL4 derived from mouse brown adipose tissue also inhibits LPL activity and promotes thermogenesis (Singh et al., 2018). The knockout of *ANGPTL4* in mice increases body fat and weight (Mattijssen et al., 2014). Some scientists believe that *ANGPTL4* and *LPL* are involved in a specific pathway. For instance, a previous study found that the overexpression of *miR-134* reduces the expression of *ANGPTL4* in the aortic tissues and peritoneal macrophages, while increasing the expression and activity of *LPL* and promoting lipid accumulation and pro-inflammatory cytokine secretion, thus accelerating the formation of atherosclerotic plaques (Ye et al., 2018). Our results are consistent with those of previous reports, suggesting that *ANGPTL4 a*nd *LPL* form a pathway in pigs, where *ANGPTL4* negatively regulates *LPL*. However, further studies are required to validate our findings.

## Conclusion

In this study, 27 co-expression modules were constructed using 12,816 genes from 12 pig preadipocyte samples via the WGCNA method. Three modules were identified as being the most critical during preadipocyte differentiation. Through WGCNA and DEG analysis, the hub gene *ANGPTL4* was identified as a potential regulator of preadipocyte differentiation and lipid metabolism. Following *ANGPTL4* inhibition, the expression of adipogenesis-related genes was also reduced, except for *LPL*. *ANGPTL4* could negatively regulate *LPL* during preadipocyte differentiation. Our findings provide a new perspective for understanding the mechanism underlying fat deposition.

## Data Availability

The datasets presented in this study can be found in online repositories. The names of the repository/repositories and accession number(s) can be found below: NCBI (accession: PRJNA509755).
