# Supplementary material for: Identification of Potential Candidate Genes From Co-Expression Module Analysis During Preadipocyte Differentiation in Landrace Pig
Source: Front Genet. 2022 Feb 1;12:753725. doi: 10.3389/fgene.2021.753725 (PMC8843850; doi:10.3389/fgene.2021.753725)
Supplement: Supplementary file 1 [file Table1.docx]

**Table S1. Primer sequences of qRT-PCR**

| Gene | Sequence (5'-3') | T_m_（℃） |
| --- | --- | --- |
| *ANGPTL4* | F: GCATGGCTGCCTGTGGTAAC | 60 |
|  | R: ATCTTGCTGTTTTGAGCCTTGA |  |
| *ACAA2* | F: TGAAATCAAGACCCTTGGGGG | 60 |
|  | R: GTACCCACAGCAAGGACACT |  |
| *ALDH2* | F: CGGTACGTACACAGCCTTCA | 60 |
|  | R: GACGAGCACTTCCCACATCA |  |
| *SLC27A1* | F: ACCCTGAAACACTGGAGGTC | 60 |
|  | R: CAGAGGCAAGCCTGGATGTA |  |
| *PPARG* | F: GATTTCTCCAGCATTTCCA | 60 |
|  | R: GCTCTTCGTGAGGTTTGG |  |
| *LPL* | F: AGCAGTTGAAGGCTGACTCC | 60 |
|  | R: GCTAGGCAGATGCCAGTTCA |  |
| *SCD* | F: TAGGAGCCAGAGGAACCAGAA | 60 |
|  | R: CACCACAAGACAGTGCATGG |  |
| *FASN* | F: GGTGGTCACAGAATGACACCT | 60 |
|  | R: GGCCTCCACTGACTCTTCAC |  |
| *GADPH* | F: ACTCACTCTTCTACCTTTGATGCT | 60 |
|  | R: TGTTGCTGTAGCCAAATTCA |  |

F: forward primer

R: reverse primer

T_m_: annealing temperature
