# Supplementary figures and images for: Identification of Potential Candidate Genes From Co-Expression Module Analysis During Preadipocyte Differentiation in Landrace Pig
Source: Front Genet. 2022 Feb 1;12:753725. doi: 10.3389/fgene.2021.753725 (PMC8843850; doi:10.3389/fgene.2021.753725)

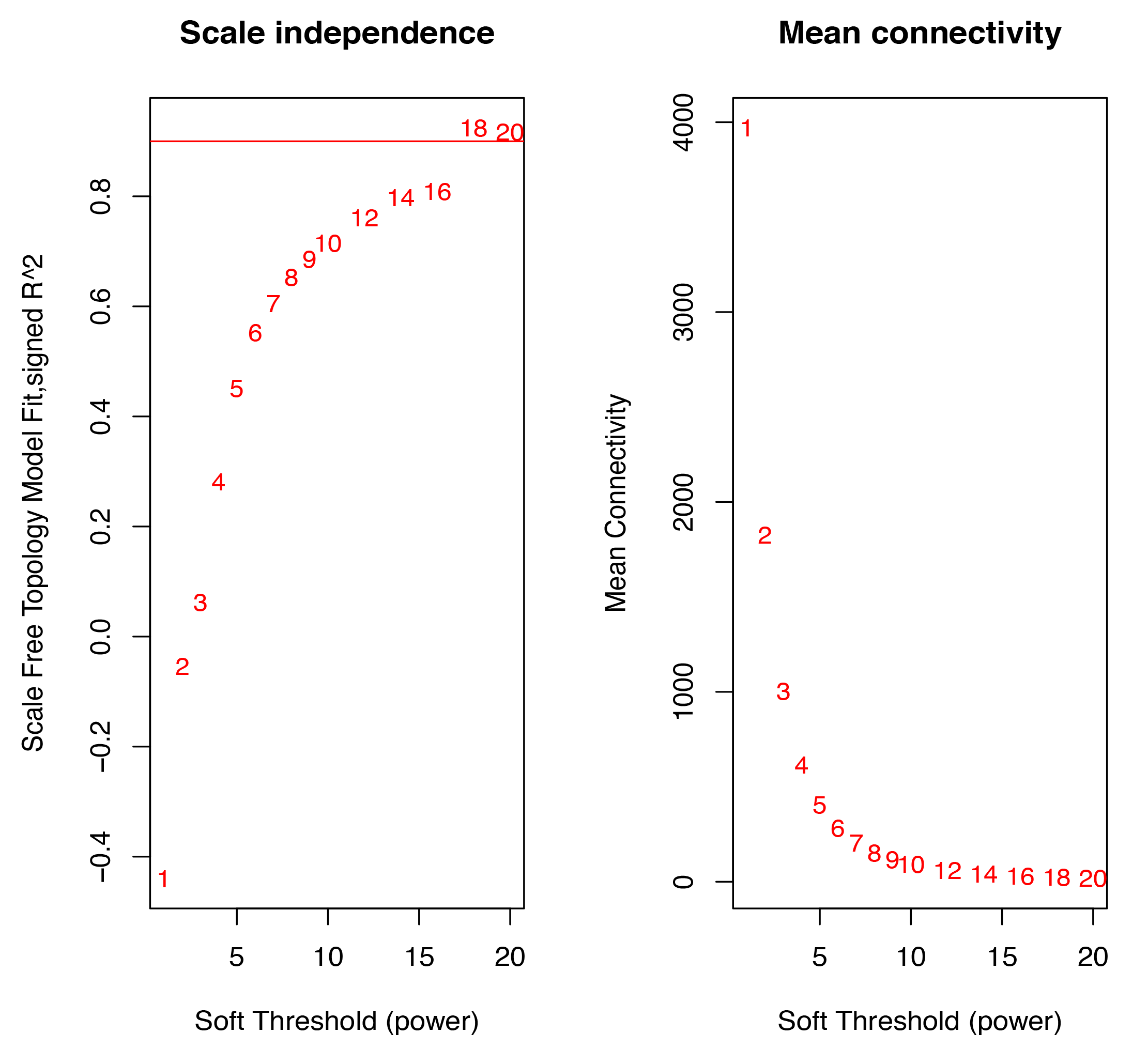

Supplement: Supplementary file 2 [file Image2.TIF]

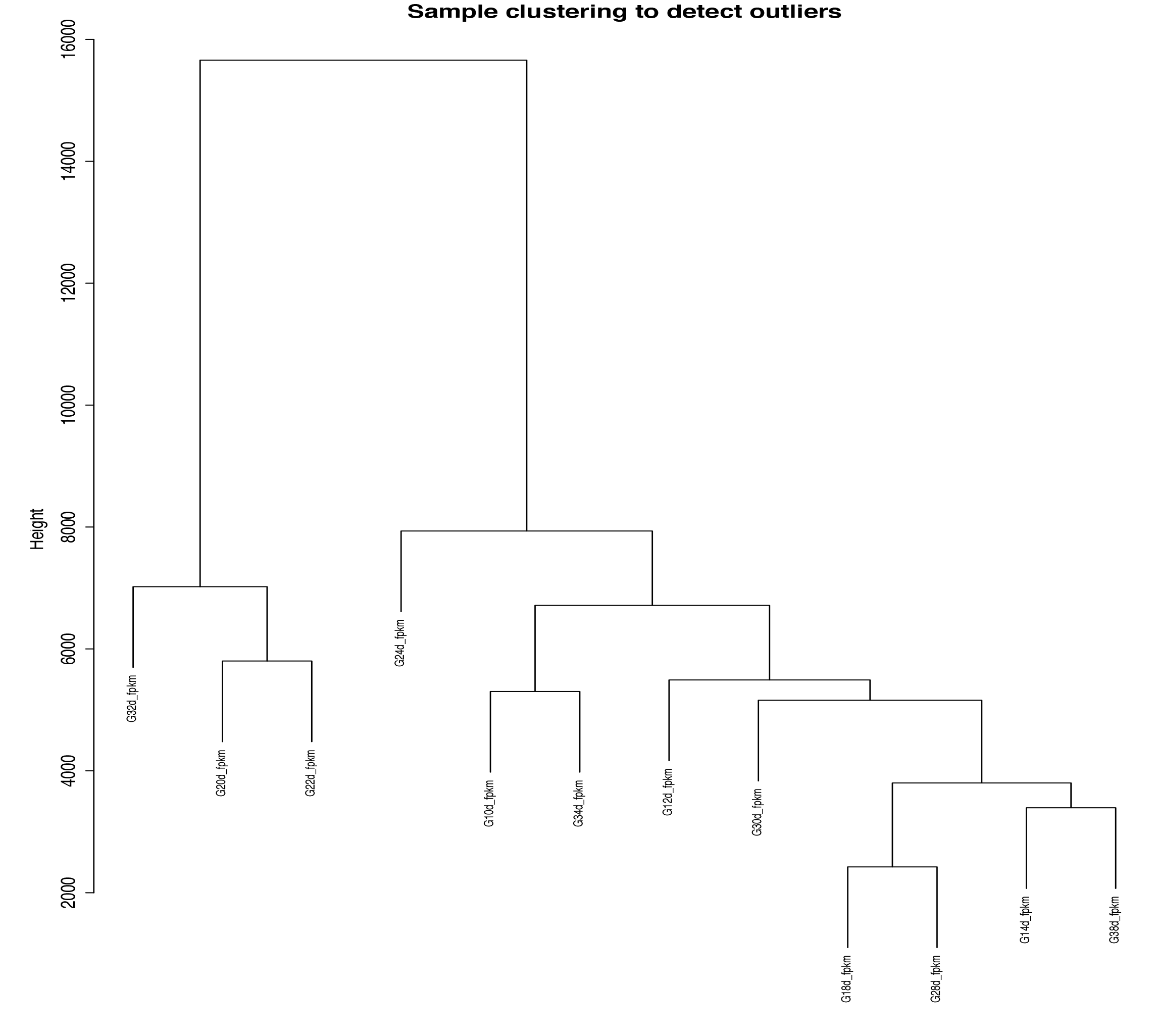

Supplement: Supplementary file 3 [file Image1.TIF]
