# Supplementary material for: Identification of Potential Candidate Genes From Co-Expression Module Analysis During Preadipocyte Differentiation in Landrace Pig
Source: Front Genet. 2022 Feb 1;12:753725. doi: 10.3389/fgene.2021.753725 (PMC8843850; doi:10.3389/fgene.2021.753725)
Supplement: Supplementary file 5 [file Table3.docx]

**Table S3. Number of genes in the constructed modules**

| **Module color** | **Gene number** | **Module color** | **Gene number** |
| --- | --- | --- | --- |
| Black | 565 | Light green | 143 |
| Blue | 1341 | Light yellow | 132 |
| Brown | 1243 | Magenta | 487 |
| Cyan | 173 | Midnight blue | 165 |
| Dark green | 80 | Orange | 51 |
| Dark gray | 66 | Pink | 513 |
| Dark orange | 43 | Purple | 441 |
| Dark red | 112 | Red | 766 |
| Dark turquoise | 67 | Royal blue | 131 |
| Green | 1133 | Salmon | 188 |
| Green-yellow | 325 | Tan | 193 |
| grey60 | 144 | Turquoise | 1676 |
| Light cyan | 160 | White | 35 |
|  |  | Yellow | 1213 |
